# Work Allocation Justice in Saudi Nursing: A Qualitative Exploration of Equity, Fairness Perceptions, and Organizational Factors Influencing Nurse Burnout and Quality of Care

**DOI:** 10.3390/healthcare14131882

**Published:** 2026-06-28

**Authors:** Hanadi Dakhilallah, Muteb Aljuhani, Waleed M. Alshehri, Thurayya Eid, Rayhanah R. Almutairi, Asrar S. Almutairi, Norah M. Alyahya, Abdulaziz M. Alodhailah

**Affiliations:** 1 Nursing Administration and Education Department, College of Nursing, Imam Mohammad Ibn Saud Islamic University (IMSIU), Riyadh 11564, Saudi Arabia; 2 Department of Community Health, Mental and Psychiatric Nursing, Imam Mohammad Ibn Saud Islamic University (IMSIU), Riyadh 11564, Saudi Arabia; 3 Department of Medical-Surgical Nursing, College of Nursing, King Saud University, Riyadh 11451, Saudi Arabia; 4 Community and Psychiatric Mental Health Nursing Department, College of Nursing, Princess Nourah bint Abdulrahman University, Riyadh 11671, Saudi Arabia; 5 Department of Community and Psychiatric Mental Health Nursing, College of Nursing, King Saud University, Riyadh 11451, Saudi Arabia

**Keywords:** work allocation justice, organizational justice, nurse burnout, job satisfaction, qualitative research, reflexive thematic analysis, Saudi Arabia, healthcare leadership, equity

## Abstract

**Background**: Perceived inequity in work allocation has well-documented consequences for nurse job satisfaction, organizational commitment, and patient safety. Despite this recognition, empirical research examining how nurses in Middle Eastern healthcare contexts conceptualize allocation justice remains limited. This qualitative study explored Saudi registered nurses’ perceptions of work allocation justice, identified the organizational factors shaping those perceptions, and examined their implications for nurse well-being and quality of care. **Methods**: A qualitative design employing reflexive thematic analysis was used, grounded in constructivism. Semi-structured individual interviews were conducted with 17 purposively sampled registered nurses from three Riyadh-based healthcare facilities. Credibility was established through data, method, and analyst triangulation; peer debriefing; and member checking (12 of the 17 participants were purposively selected for member checking to represent the full range of experiences; 11 of those 12 confirmed thematic plausibility). Thematic saturation was reached at interview 13. **Results**: Six interconnected themes emerged: (1) Understanding Work Justice; (2) Personal Experiences; (3) Influencing Factors; (4) Psychological and Professional Impacts; (5) Institutional and Administrative Support; and (6) Future Perspectives. Participants define fairness as contextual equity and relational respect rather than simple numerical equality. Inequities were driven by staffing constraints, leadership styles, and policy gaps, leading to burnout and reduced organizational commitment. **Conclusions**: Saudi nurses experience meaningful allocation inequities arising from structural constraints, leadership variability, and the absence of written allocation policies. Addressing these inequities requires coordinated action on policy transparency, objective criteria, and psychologically safe communication channels. These findings provide contextually grounded, evidence-informed guidance for developing equitable nursing work environments that support workforce retention and patient safety.

## 1. Introduction

Work allocation justice (defined as the perception that patient assignments, responsibilities, and workload are distributed fairly among nursing staff) represents a critical yet frequently overlooked dimension of clinical practice environments [1,2]. Growing evidence from organizational psychology and healthcare management demonstrates that perceived inequity in task distribution profoundly influences nurse stress responses, job satisfaction, burnout trajectories, and ultimately patient outcomes [3,4]. Despite this recognition, nurses’ lived experiences of allocation justice in Middle Eastern healthcare contexts remain inadequately examined [5,6]. This gap is particularly notable in Saudi Arabia, where nursing has undergone substantial professionalization yet continues to face significant workforce challenges, including high burnout rates and retention difficulties.

### 1.1. Conceptual Foundation

Organizational justice theory provides a framework for understanding how fairness perceptions shape employee attitudes, behaviors, and outcomes [7]. It comprises three dimensions: distributive justice (fairness of outcomes), procedural justice (fairness of decision-making processes, including transparency and consistency), and interactional justice (quality of interpersonal treatment and respect) [8,9]. In nursing, work allocation justice spans all three: equitable patient assignments, transparent and consistent decision-making with opportunities for input, and respectful recognition of nurses’ expertise and efforts. While evidence from high-income countries highlights the importance of these dimensions, their operationalization, relative importance, and cultural framing in non-Western healthcare systems remain underexplored [1,10]. Examining justice perceptions across diverse organizational and cultural contexts can both extend theory and inform locally relevant practice interventions.

### 1.2. The Saudi Arabian Healthcare Context

Saudi Arabia represents an important context for studying work allocation justice [11]. The Kingdom’s investments in healthcare infrastructure, nursing education, and workforce Saudization have created complex workplaces where allocation practices mix hierarchical, merit-based, and hybrid approaches [12,13]. Contextual factors, including diverse facility types, cultural values like respect for authority, concepts such as adl (justice) and wasta (relationship influence), a largely international nursing workforce, and national priorities for patient safety and retention, shape how nurses perceive and respond to allocation inequities [14,15]. Despite the documented impact of these factors on care quality and workforce stability, research specifically addressing allocation justice in Saudi nursing remains limited. The international literature is dominated by high-income anglophone contexts, constraining the availability of culturally relevant guidance for local policy and practice.

### 1.3. Study Aims and Objectives

This qualitative study explored Saudi nurses’ perceptions of work allocation justice, examining how they define fairness, experience allocation practices in daily work, identify organizational and contextual influences, and propose improvements. The objectives were to: (1) examine nurses’ conceptualizations of allocation justice and fairness dimensions; (2) document experiences of equitable and inequitable allocation and associated emotional and professional responses; (3) identify organizational, leadership, and structural factors shaping fairness perceptions; (4) clarify the psychological, professional, and care-related consequences of perceived inequity; (5) assess the adequacy of institutional policies and communication processes; and (6) capture nurses’ evidence-informed recommendations for organizational and policy reform.

## 2. Materials and Methods

### 2.1. Study Design and Epistemological Positioning

A qualitative design using reflexive thematic analysis within a constructivist framework examined nurses’ experiences of work allocation justice. Constructivism, which regards knowledge as co-constructed through researcher–participant interaction [16,17], is well suited to justice research, as fairness perceptions are shaped by cultural, relational, and personal interpretations. Thematic analysis provided a systematic yet flexible method to identify patterns while remaining close to participant meaning, allowing justice conceptualizations to emerge inductively from Saudi nursing perspectives. Semi-structured interviews were treated as co-constructed dialogs, and coding was interpretive, with multiple researchers independently coding, discussing differences, and reaching consensus. The resulting themes represent one credible interpretive account, acknowledging that alternative readings of the data remain possible. The credibility procedures reported in Section 2.11 (triangulation, member checking, peer debriefing, and reflexivity documentation) were selected as complementary strategies within this reflexive, constructivist framework following [18] Lincoln and Guba not as substitutes for its interpretive philosophy; independent coding served consensus-building rather than reliability testing, and member checking validated interpretive plausibility rather than factual accuracy [18].

### 2.2. Setting and Participant Selection

The study was conducted across three healthcare facilities in Riyadh, Saudi Arabia, selected to provide diversity in governance models, funding structures, and practice contexts. The facilities included two university-affiliated teaching hospitals (one government, one private) and one specialized medical center serving urban populations. All three facilities employ mixed nursing workforces comprising Saudi nationals and internationally trained nurses, reflecting the contemporary Saudi healthcare system composition. These settings were selected to maximize organizational diversity of perspectives and to support the analytic transferability of findings to broadly similar Saudi healthcare contexts. In all three facilities, daily work allocation is managed at the unit level by the charge nurse or shift supervisor, who assigns patients to nurses at the start of each shift. Assignments are made verbally and are not formally documented beyond the unit whiteboard in most cases. No standardized written allocation policy was in place at any of the three sites at the time of data collection; assignment decisions were at the discretion of the shift supervisor, informed by patient census, nurse availability, and the supervisor’s judgment about individual nurse capacity. This procedural context is central to interpreting participants’ perceptions of fairness: the absence of transparent, documented criteria meant nurses could not verify the basis for any given assignment, which several participants identified as the primary source of perceived inequity.

### 2.3. Purposive Sampling Strategy

Purposive sampling was employed with strategic variation across demographic and professional dimensions to ensure that the sample captured heterogeneity of perspectives within the Saudi nursing context. Sampling criteria included: age (targeting variation across career stages, from early to senior career), years of nursing experience (from 2–5 years through 15+ years), educational preparation (Diploma, Bachelor’s, Master’s degrees), workplace facility type (government, private, specialized), clinical specialty (high-acuity intensive care, acute medical–surgical units, emergency departments, pediatrics, operating theatre), and shift patterns (day, evening, night, rotating). This sampling approach ensured that the final sample reflected professional diversity across the Saudi nursing context and supported analytic credibility by incorporating varied perspectives and experiences of work allocation justice.

### 2.4. Inclusion and Exclusion Criteria

Inclusion criteria were: (1) current employment as a registered nurse or specialist nurse at one of the three participating healthcare facilities; (2) Saudi nationality; (3) minimum of 2 years’ nursing experience; (4) fluency in Arabic or English sufficient for interview participation; and (5) voluntary informed consent. Exclusion criteria were: (1) nurses in administrative or management roles, to focus on direct care provider perspectives; (2) nurses with less than 2 years’ experience, to ensure substantive workplace exposure; and (3) acute illness or inability to participate due to medical or personal circumstances. Managers were excluded to maintain focus on frontline nurse perspectives; their views on allocation rationales would require a separate study design. Eligible participants were identified through ward managers at each facility, who distributed study information sheets to nursing staff meeting the inclusion criteria; interested nurses contacted the principal investigator directly to express willingness to participate. Snowball referral was used as a secondary recruitment strategy where initial participants recommended eligible colleagues.

### 2.5. Saturation Assessment and Sample Adequacy

Thematic saturation, operationalized a priori as the emergence of fewer than five new codes per interview with concurrent stability of subthemes across all six domains, served as the criterion for sample adequacy. This threshold was reached by interview 13; interviews 14 and 15 confirmed data redundancy. Interviews 16 and 17 had been scheduled prior to the formal saturation assessment and were retained to maximize experiential diversity across facility types. This approach aligns with established qualitative standards that prioritize information richness and saturation over predetermined sample sizes [19].

### 2.6. Data Collection Procedures

Data were collected through semi-structured individual interviews guided by a 20-item interview guide developed through iterative literature review, team consultation, and pilot testing with two nurses. Questions covered six domains: definitions of work allocation justice, experiences of equitable/inequitable allocation, influencing factors, psychological and professional impacts, institutional support and communication, and improvement suggestions. This flexible format enabled in-depth exploration of individual experiences while maintaining consistency across interviews. Interviews were conducted by the principal investigator and two trained research assistants between 15 December 2025 and 10 February 2026 (ethical approval was granted on 15 December 2025; recruitment commenced following receipt of approval, consistent with King Saud University IRB practice), lasting between 45 and 90 min (mean: 63 min; total: approximately 23 h), in private rooms within each facility, and were audio-recorded and transcribed verbatim with participant consent.

### 2.7. Translation Procedures and Language Management

Interviews were conducted in participants’ preferred language (71% Arabic, 29% English). Arabic interviews were transcribed verbatim and translated into English by two independent bilingual team members. To ensure semantic fidelity, 25% of Arabic transcripts underwent back-translation by a third independent translator, with discrepancies resolved through team discussion. Particular care was taken with culturally and philosophically nuanced terms central to justice discourse, namely adl (justice/fairness), haq (right/entitlement), ihsan (excellence beyond duty), and takaful (mutual solidarity), with all interpretive translation decisions documented and subjected to team review during analysis.

### 2.8. Field Notes and Contextual Documentation

Field notes capturing contextual observations, interviewer reflections, emerging analytical impressions, and non-verbal participant cues were recorded immediately following each interview. These notes documented interview setting characteristics, visible emotional responses, interruptions, participant engagement, and interviewer observations that enriched interpretation beyond the recorded transcript. Field notes were incorporated into reflexivity discussions during analysis and contributed to comprehensive audit trail documentation.

### 2.9. Research Team Composition and Reflexivity

The research team included four nurse academics with expertise in qualitative methodology, organizational behavior, nursing leadership, and Middle Eastern healthcare contexts. The principal investigator holds a doctoral qualification in nursing science, with over eight years of qualitative research experience and five years of direct clinical practice in Saudi Arabia. This background provided contextual familiarity with the study setting while simultaneously requiring rigorous management of positionality and potential analytical bias.

Reflexivity was embedded throughout the study. Prior to data collection, all team members completed bracketing exercises to document pre-existing assumptions and expectations regarding organizational justice and fairness. Reflexive memos were maintained throughout the analytical process to capture evolving interpretations, analytical challenges, and moments when assumptions required interrogation. These reflections were discussed in regular team debriefings to ensure multiple perspectives informed the analysis.

A detailed Reflexivity Statement outlines team members’ positionality, documented biases, and how the insider–outsider composition strengthened interpretive rigor and transparency.

### 2.10. Data Analysis Procedures

Thematic analysis proceeded through six iterative, constructivist-informed phases, recognizing coding and theme development as inherently interpretive processes.

Phase 1: Familiarization. Analysts independently read initial transcripts in full, recording impressions and preliminary patterns to build deep data familiarity.

Phase 2: Initial Coding. Line-by-line inductive coding was conducted, grounded in participant language. Coding memos documented definitions and refinements. Approximately 180 initial codes emerged across the first 12 interviews. Thematic saturation was reached by interview 13; interviews 14 and 15 confirmed data redundancy. Interviews 16 and 17, which had been scheduled prior to the formal saturation assessment, were retained to maximize experiential diversity across facility types, consistent with the approach described in Section 2.5.

Phase 3: Searching for Themes. Related codes were grouped into candidate themes and subthemes based on conceptual similarity. Thematic mapping supported team discussion and analytic transparency.

Phase 4: Reviewing Themes. Themes were evaluated for internal coherence and distinctiveness. Underdeveloped themes were merged or refined, resulting in six major themes and 18 subthemes with strong data support.

Phase 5: Defining and Naming Themes. Themes were clearly defined, bounded, and named, with detailed descriptions and exemplar quotations linked to the research objectives.

Phase 6: Producing the Report. Themes were organized into a coherent narrative integrating participant quotations and analytic interpretation to present a comprehensive account of nurses’ experiences of work allocation justice. Verbatim quotations attributed to alphanumeric participant codes (P1–P17) are used throughout the Results to exemplify each theme and subtheme.

### 2.11. Software and Analytical Rigor

NVivo 12 (QSR International, Melbourne, Australia) software was used for data management, coding organization, memo documentation, and audit trail preservation, supporting analytical organization and transparency without directing the analytic process. Analysts maintained independence in coding processes; software served as a repository and organizational tool rather than a decision-making mechanism.

#### Credibility and Trustworthiness

Rigor was enhanced through multiple strategies aligned with qualitative research standards [17,18]:

1. Triangulation: Combined data source (diverse nurses across three facilities), method (interviews plus field notes), and analyst triangulation (independent coding with team discussions).
2. Member Checking: Twelve of the 17 participants were purposively selected for member checking to represent maximum variation across facility types, career stages, and themes. Of these 12, eleven reviewed the thematic summaries (provided in both Arabic and English), confirmed alignment with their experiences, and suggested refinements, such as adding a subtheme on informal peer-based workload balancing. This denominator reflects deliberate purposive selection for the validation step, not attrition from the full sample.
3. Peer Debriefing: An external qualitative expert reviewed coding, theme development, and interpretive logic to ensure findings were grounded and credible.
4. Audit Trail: All analytic decisions, memos, maps, debriefing notes, and feedback summaries were systematically documented for transparency.
5. Reflexivity Documentation: Bracketing exercises, reflexive memos, and team debriefings were maintained to acknowledge and manage researcher positionality throughout the study.

### 2.12. Ethical Considerations

Ethical approval was obtained from King Saud University’s IRB (KSU-HE-25-1418). This central approval covered all three participating facilities; site-level administrative permissions were additionally obtained from the nursing directorate of each facility prior to recruitment. The private hospital operates under a research governance framework that recognizes King Saud University IRB approval, and written institutional permission was obtained from that site separately. Participants provided written informed consent, with clear explanations of voluntary participation, withdrawal rights, confidentiality, and study procedures in Arabic or English. To protect confidentiality, participants were assigned alphanumeric codes (P1–P17), identifying information was removed from transcripts, and electronic data were stored on encrypted, password-protected servers. Aggregated thematic findings, presented at the institutional rather than unit level and containing no identifying participant or unit-level information, were shared with facility research coordinators to support evidence-informed quality improvement. No individual participant data, unit-specific details, or quotations were disclosed in any form that could enable re-identification.

### 2.13. Data Availability

Due to the sensitive nature of qualitative interview data and ethical constraints protecting participant confidentiality, original transcripts are not publicly available. However, researchers may request anonymized data subsets (coding framework, thematic summaries with exemplar quotations, selected thematic excerpts) or discussion of the thematic coding framework by contacting the corresponding author. Original audio recordings were destroyed after transcription by study protocol and participant consent agreement.

## 3. Results

### 3.1. Participant Characteristics

Table 1 presents the demographic and professional characteristics of the 17 study participants. The sample included nurses aged 25 to 49 years (mean 34.5 years, SD 7.2 years). Educational qualifications reflected the diversity of Saudi nursing preparation pathways: nine participants (53%) held nursing diplomas, five (29%) held Bachelor of Science degrees in nursing, and three (18%) held master’s degrees. Years of nursing experience ranged from 2 to 15+ years: 29% had less than 5 years’ experience, 47% had 5–10 years’ experience, and 24% had more than 10 years’ experience. Participants worked across diverse clinical specialties: intensive care (29%), medical–surgical (24%), emergency (18%), pediatrics (12%), and operating theatre (18%). Participants were drawn from government hospitals (41%), private hospitals (35%), and specialized medical centers (24%). Shift patterns included day shifts (41%), evening shifts (29%), night shifts (24%), and rotating shifts (6%). Fifteen participants (88%) reported direct personal experience of allocation inequities; two (12%) reported generally equitable allocation within their unit.

This breadth of demographic representation strengthened analytic credibility and supported transferability by incorporating perspectives across career stages, educational preparation levels, and organizational contexts within the Saudi healthcare system.

### 3.2. Themes and Subthemes Overview

Six interconnected themes emerged from the thematic analysis, each addressing a distinct facet of work allocation justice and its implications. Table 2 presents an overview; detailed descriptions follow.

**Figure 1:**
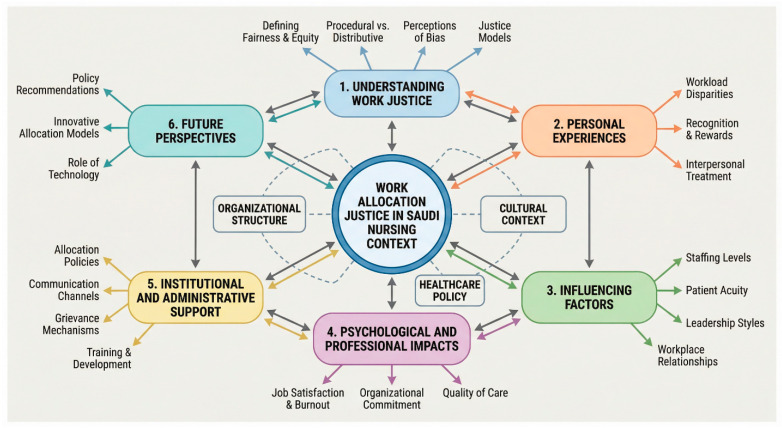
Conceptual Framework of Work Allocation Justice in the Saudi Nursing Context.

Figure 1 illustrates the multi-dimensional factors influencing work allocation and equity within Saudi Arabian healthcare settings. The model integrates six primary domains: Work Justice, Personal Experiences, Influencing Factors, Psychological and Professional Impacts, Institutional Support, and Future Perspectives, mediated by organizational structure, cultural context, healthcare policy, and nursing leadership. Figure 1 serves a conceptual purpose distinct from Table 2: whereas Table 2 enumerates themes and subthemes with descriptive text, Figure 1 depicts the relational structure and mediating pathways between domains. Authors should verify that all domain labels and sub-elements are fully legible in the final published version, particularly those within and below the Psychological and Professional Impacts domain.

#### 3.2.1. Theme 1: Understanding Work Allocation Justice

Participants articulated multidimensional conceptions of work allocation justice that extended well beyond simplistic notions of numerical equality. Three subthemes captured the complexity of these conceptualizations.

##### Subtheme 1A: Fairness as Contextual Equity Rather than Identical Equality

Nurses define fairness primarily through relational and contextual lenses rather than strictly proportional distribution. P11 explained: “Justice in distribution means that the workload is allocated based on each person’s capacity and the complexity of patients they are assigned. It’s not just about the numbers—it’s about understanding that some nurses are newer, or some have more pressure at home, or some units are more exhausting.” Similarly, P3 articulated: “We need to think about what is fair, not just equal. Some patients need more time and care. A fair distribution considers these differences and the individual situation of each nurse.”

This understanding clearly distinguished between equality (identical distribution regardless of circumstance) and equity (distribution proportionate to legitimate differences in need, capacity, and circumstance). Participants demonstrated nuanced reasoning about contexts in which identical task allocation would be unjust: for example, assigning experienced and novice nurses equal patient loads without accounting for competency differences, or ignoring a nurse’s significantly elevated personal burden when setting their workload.

##### Subtheme 1B: Procedural Justice and Transparent Decision-Making

Beyond the distributive dimension, participants consistently emphasized the importance of transparent, consistent, and rationally defensible processes for allocation decisions. P7 observed: “It’s not just whether the distribution is fair, but how decisions are made. Are the rules the same for everyone? Do managers explain why assignments are given the way they are? When I don’t understand the logic, I feel that something is unfair, even if the actual assignment might be okay.”

Participants reported significant distress when allocation decisions appeared arbitrary or inconsistently applied. The absence of transparent criteria meant that even numerically balanced distributions generated strong perceptions of inequity when the reasoning behind them was opaque. P14 articulated this dynamic: “What bothers me most is not knowing why I got this assignment while my colleague got another. If there were clear rules and the manager explained it, I could accept it. But the uncertainty makes me think someone is being favored.”

##### Subtheme 1C: Multiple Justice Frameworks and Their Conflicts

Participants articulated distinct implicit justice frameworks that informed their allocation evaluations, sometimes generating tension when different frameworks were applied inconsistently. Three primary frameworks emerged:

Needs-based justice: Some participants emphasized support for struggling colleagues, articulated as: “Those who have the most difficult patients or who are struggling should get lighter loads sometimes to help them succeed” (multiple participants, e.g., P1, P8, P15). This framework prioritizes equity by acknowledging different capacities and circumstances.

Meritocratic justice: Others emphasized recognition of competence and capability, articulated as: “Those who are more competent should have more responsibility; it’s a way of recognizing their abilities” (multiple participants, e.g., P3, P6, P9). This framework rewards demonstrated skill and creates incentives for professional development.

Seniority-based justice: Still others emphasized experience and tenure, articulated as: “Those with more experience should have a bit more responsibility because they can handle it and should be developing the less experienced staff” (multiple participants, e.g., P5, P12, P16). This framework values institutional knowledge and mentorship.

When observed allocation practices reflected one framework while a nurse believed another should prevail, significant injustice was perceived. P6 articulated this tension acutely: “I studied hard, got my degree, and I’m a good nurse. But the senior nurses who have just diplomas get preference for the best patients because they have been here longer. That doesn’t feel just to me.” This statement reveals conflict between meritocratic expectations (educated, competent nurses should receive preferred assignments) and seniority-based practices (tenure determines allocation regardless of education or demonstrated ability).

#### 3.2.2. Theme 2: Personal Experiences with Allocation Inequities

Fifteen of 17 participants reported direct experiences of perceived work allocation inequities. The two participants who did not report personal inequities attributed this to transparent and consistent charge nurses within their units; their accounts nonetheless corroborated the patterns described by the majority. For the majority, inequitable allocation was recurrent and emotionally salient, often spanning multiple clinical settings and career stages. Two subthemes captured the substance and emotional impact of these experiences.

##### Subtheme 2A: Workload Disparities and Differential Treatment

Participants described concrete situations wherein workload appeared unequally distributed. P2 recounted: “Some days I’m assigned five critical patients alone while another nurse-doing the same job, same shift-gets two patients. When I asked why, I was told ‘that’s just how it worked out.’ It happened repeatedly with the same people getting fewer patients. I felt like I was being punished for something.” P9 added: “I noticed that nurses who are friends with the charge nurse get lighter assignments. When I mentioned it, nothing changed. It made me question whether my skills were valued or if I was just being taken advantage of.”

Participants identified four recurring patterns in their experiences of disparate allocation:

- Relationship-based allocation: Some reported that assignment distribution reflected informal relationships or social proximity to managers rather than merit or legitimate patient care factors. This pattern generated particular resentment because it signaled that collegial networks, rather than competence or need, determined workload.
- Seniority-based disparities: Several participants observed that particular career stages or tenure levels received preferential treatment independent of current capacity or patient need.
- Specialty or shift burden: Some noted that particular specialties (e.g., intensive care) or shifts (e.g., night shifts) bore disproportionate allocation burdens as organizational norms rather than by necessity.
- Punitive allocation: Several participants perceived that raising concerns about allocation, safety, or managerial decisions was followed by heavier workload assignments, suggesting a pattern of punitive redistribution.

These experiences generated sustained resentment and demoralization. P5, with 15+ years of experience, stated poignantly: “After all these years, I expected better. I see newer nurses getting choice assignments. I try not to be bitter, but it’s hard when you’ve sacrificed so much and feel like you’re not respected.” This statement captured the accumulated emotional toll of perceived long-term inequity and the erosion of organizational commitment it produced.

##### Subtheme 2B: Inequitable Recognition and Appreciation

Beyond numerical workload disparities, participants described significant inequities in acknowledgment and appreciation of their contributions. P4 observed: “Some nurses get public recognition when they do good work. Others, including myself, do the same quality work but never hear a word of thanks from the manager. The ones who get recognized seem to be the ones the manager personally likes.”

This inequitable distribution of recognition compounded the distress of allocation inequity. When nurses felt their clinical contributions and professional efforts were unacknowledged or selectively appreciated based on personal relationships rather than performance, they interpreted allocation decisions as further evidence of broader undervaluation. P13 articulated the cumulative impact: “It’s not just the workload itself. It’s feeling invisible. Your efforts don’t matter. That’s when you start thinking about leaving.”

#### 3.2.3. Theme 3: Organizational and Contextual Factors Influencing Allocation Justice

Participants identified a range of organizational and contextual factors that shaped both allocation practices and justice perceptions.

##### Subtheme 3A: Staffing Levels, Resource Constraints, and Transparency

When adequate staffing was unavailable, most participants understood that allocation might not perfectly balance workload burdens across the team. P1 noted: “When we’re short-staffed, someone has to carry more. I understand that. But I’d appreciate the manager being honest about it-‘we don’t have enough people, so today I need you to take an extra patient, and I’m sorry’-rather than pretending it’s a fair distribution.”

However, participants perceived that chronic understaffing was sometimes inappropriately used to justify systemic inequities that transcended resource constraints. P8 observed: “Staffing is tight, sure. But I’ve seen situations where, if a nurse the manager liked was struggling, they’d call someone on or shift assignments. But when it was someone, the manager didn’t prefer, they’d just say ‘we don’t have coverage.’ This pattern suggested that resource constraints were at times genuine barriers to equitable allocation, yet at other times were invoked as post hoc justifications for decisions that in practice reflected managerial preferences.

##### Subtheme 3B: Patient Acuity Assessment, Objectivity, and Consistency

Participants recognized that patient conditions varied legitimately and should appropriately inform assignment distribution. However, they questioned whether acuity assessments were conducted consistently and objectively. P10 remarked: “The patients I get assigned are often quite complex, but I see other nurses getting patients described as high acuity who are actually stable. I wonder if the acuity classifications are reliable or if they’re just used to justify whatever assignment the manager wants.”

This concern reflected a broader uncertainty about whether acuity classification tools served as genuine guides to equitable distribution or as post hoc justifications for decisions driven by other considerations.

##### Subtheme 3C: Leadership Styles and Decision-Making Processes

Participants described substantial variation in managers’ approaches to allocation. Some managers solicited input, explained rationales, and adjusted assignments when concerns were raised, generating trust even when distributions were not perfectly equal. Others made allocations unilaterally, offered no justification, and dismissed concerns—generating distrust and perceived inequity regardless of the objective fairness of distributions.

P12 contrasted with two managers: “My previous manager would say, ‘Here’s your assignment and here’s why I made this choice. Do you see any problems with it?’ Even if I didn’t like it, I felt heard. My current manager just assigns and leaves. Same hospital, same policy, but completely different experience.” This distinction highlighted that justice perceptions are substantially determined by process quality and relational respect, not merely by objective outcomes.

##### Subtheme 3D: Informal Peer-Based Balancing

Beyond formal allocation systems, participants described informal mechanisms through which nurses collectively balanced workload inequities. P11 explained: “Sometimes when the allocation is unfair, the nurses who have lighter loads will help those who are overwhelmed. We communicate quietly and help each other. It’s not official, but it’s how we survive.” This informal peer-based support sometimes mitigated the worst effects of inequitable formal allocation but did not address the underlying injustice or reduce the emotional toll.

#### 3.2.4. Theme 4: Psychological and Professional Impacts

Perceived work allocation injustice produced significant psychological and professional consequences for frontline nurses.

##### Subtheme 4A: Job Satisfaction and Burnout

Participants who felt allocation was unjust reported lower job satisfaction and higher burnout symptoms. P15 stated: “I used to love this job. But the unfairness has worn me down. I feel exhausted not just physically but emotionally. I question why I’m working so hard when it’s not appreciated or acknowledged. I think about quitting regularly.”

Several participants articulated the pathway from injustice to burnout explicitly. When workload felt inequitably distributed, nurses absorbed additional effort to manage the overload, generating physical and emotional fatigue. When those efforts went unrecognized, motivation and professional meaning eroded progressively. Over time, this trajectory produced depersonalization (emotional detachment from patients) and reduced personal accomplishment, corresponding to the core dimensions of burnout identified in occupational health research [20].

##### Subtheme 4B: Organizational Commitment and Retention

Perceived allocation injustice was linked to reduced organizational commitment and intention to leave. P3 stated: “This hospital treated me well when I first arrived. But as I’ve seen how unfairly the allocation is done and how certain people are favored, I’ve lost faith in the institution. I’m looking for another job where I’ll be treated fairly.”

Several participants explicitly connected allocation injustice to turnover intentions. P9 noted: “I’ve been here five years and I’m good at what I do. But because the allocation isn’t fair and I’m not one of the favored ones, I’m considering leaving. That’s sad because I could have contributed so much more if I felt valued.”

##### Subtheme 4C: Quality of Care Implications

Participants articulated specific concerns about how perceived allocation inequity compromised the quality of patient care. P11 explained: “When I’m overwhelmed with inequitable assignments, I’m not as attentive to details. I’m rushing. I might miss something important. That worries me for patient safety. And when I see colleagues with lighter loads who have time to chat, while I’m drowning-that doesn’t seem right for patient care.”

This finding aligns with the broader literature linking nurse stress, attentional capacity, and clinical error rates. Nurses operating under perceived injustice and resulting Notably, concerns about care quality were expressed not only by nurses carrying heavy allocations but also by those who recognized that their colleagues were overburdened. P16 observed: “When I see a colleague struggling because she has five critical patients and I have two, I want to help, but sometimes I cannot. That inequality is bad for all the patients on the ward, not just hers.” This collective perspective reinforces that allocation inequity functions as a unit-level, not merely individual-level, patient safety risk.

#### 3.2.5. Theme 5: Institutional and Administrative Support

This theme documents the inadequacy of formal institutional structures and processes in supporting fair allocation.

##### Subtheme 5A: Absence of Clear Allocation Policies

Most participants reported the absence of clear, written policies governing work allocation. P7 stated: “I’ve never seen a written policy about how patients are assigned to nurses. Decisions seem to be made on the fly by whoever is in charge that day.”

When policies did exist, participants questioned their implementation. P14 remarked: “There’s supposedly a policy, but it’s not followed consistently. What’s written doesn’t match what I see happen. That makes me question whether the policy is real or just for show.”

The absence of transparent policies created an information void that participants filled with suspicion and attribution of unfair motives. Without explicit criteria and processes, nurses defaulted to wondering whether allocation reflected favoritism, discrimination, or arbitrary preference rather than professional judgment or organizational necessity.

##### Subtheme 5B: Limited Communication Channels and Psychological Safety

Participants described limited opportunities to voice concerns about allocation. While most facilities had formal grievance mechanisms, participants reported low confidence in these channels. P6 stated: “There’s supposed to be a way to file complaints, but everyone knows that if you complain, you’ll be marked as difficult. I don’t feel safe using those channels.”

P13 added: “I tried raising the issue with my manager once. She just said, ‘That’s how it is. If you don’t like it, there are other jobs.’ I never brought it up again.” This experience reflected broader patterns of limited psychological safety around allocation concerns and the perceived risks of voice behavior.

##### Subtheme 5C: Grievance Mechanisms and Leadership Response to Concerns

Even when concerns were voiced, participants felt they were dismissed or resulted in retaliation. P5 stated: “When I raised concerns about unfair allocation, nothing changed. But I felt like I was labeled as someone who complains, and subsequent assignments became harder. It felt like punishment for speaking up.”

This pattern, termed a “retaliation chill,” discouraged future voice behavior and reinforced perceptions of injustice embedded within institutional structures. When organizations respond to fairness concerns with punishment rather than inquiry, they institutionalize silence and disengagement around inequity. Subthemes 5a through 5c together reveal a systemic institutional failure operating at three mutually reinforcing levels:

#### 3.2.6. Theme 6: Future Perspectives

Participants offered substantive, experience-grounded suggestions for improving work allocation justice, organized across three subthemes.

##### Subtheme 6A: Innovative Allocation Models

Several participants suggested systematic approaches to allocation that would constrain subjectivity and distribute burden more equitably over time. P4 proposed: “What if we had a rotation system so that every nurse gets a mix of patient types and complexity levels? Everyone would have hard shifts and easier shifts, but over time it would balance out. That would be fairer.”

P10 suggested: “We could use an objective scoring system for patient acuity and complexity, then pair that with individual nurse capabilities. The computer would help assign fairly, and the manager couldn’t override it based on favoritism.”

These proposals reflected participants’ desire for allocation systems that limit subjective judgment and promote equitable outcomes over time, while remaining sensitive to legitimate variations in patient acuity and individual nurse capability.

##### Subtheme 6B: Role of Technology

Several participants saw potential in digital tools for allocation decision support. P7 stated: “If we had software that tracked which nurse had which patients and made assignments transparent, it would be harder for unfair patterns to develop unseen.” However, others cautioned: “Technology alone won’t fix it. We need the will to be fair and managers who care about justice. Technology just makes it visible.” (P10)

This nuanced view acknowledged that technology could support fair allocation by increasing transparency and reducing subjectivity but recognized that technology was not a substitute for organizational commitment to fairness and leadership accountability.

##### Subtheme 6C: Policy and Organizational Culture Change

Participants emphasized the need for institutional commitment to fairness transcending any single intervention. P14 proposed: “We need clear policies written down and communicated to everyone. We need managers who explain their decisions. We need to feel safe raising concerns. And we need to see that when concerns are raised, something actually changes.”

P2 articulated a broader vision: “This is about respect. It’s about the organization saying, ‘We value each nurse and we’re committed to treating everyone fairly.’ That has to come from the top. Leaders have to model it and demand it. Then people will follow.” This perspective recognized that sustainable change in allocation justice required cultural transformation alongside policy and procedural changes.

## 4. Discussion

### 4.1. Overview of Key Findings

The findings indicate that Saudi nurses’ experiences of work allocation justice are broadly consistent with established organizational justice theory [7] while extending it in culturally and contextually specific directions relevant to healthcare organizations across the Middle East and comparable non-Western settings. Six interconnected themes emerged from the analysis. Theme 1 (Understanding Work Justice) established that participants conceptualized fairness as contextual equity informed by multiple, sometimes competing, justice frameworks. Theme 2 (Personal Experiences) documented concrete experiences of workload disparities and inequitable recognition that generated sustained emotional distress. Theme 3 (Influencing Factors) identified staffing constraints, patient acuity inconsistencies, leadership variability, and informal peer-balancing as the organizational conditions shaping allocation practices. Theme 4 (Psychological and Professional Impacts) traced pathways from perceived injustice to burnout, reduced organizational commitment, and compromised care quality. Theme 5 (Institutional and Administrative Support) revealed a systemic institutional failure encompassing absent policies, unsafe reporting channels, and active suppression of voice. Theme 6 (Future Perspectives) captured nurses’ practical recommendations for rotation systems, technology-supported allocation, and cultural change. The Discussion that follows situates each thematic cluster within the theoretical and empirical literature and identifies implications for practice, leadership, and policy.

### 4.2. Multidimensional Operationalization of Justice in Healthcare Contexts

Participants’ articulation of fairness encompassed distributive, procedural, and interactional dimensions consistent with Greenberg’s [7] foundational organizational justice framework. Nurses evaluated allocation decisions not merely by outcomes (whether workload was numerically balanced) but equally by processes (whether decisions were transparent, consistent, and logically defensible) and interactions (whether they received respectful communication and recognition). This finding confirms the theoretical utility of multidimensional justice frameworks in healthcare contexts and indicates that interventions targeting only distributional equity will be insufficient when procedural and relational dimensions remain deficient.

Moreover, participants’ conceptualization of fairness as equity rather than strict equality, which acknowledges that context-appropriate allocation must account for patient complexity, individual capability, and personal circumstance, reflects a more sophisticated justice reasoning than simple proportionality. This is consistent with recent scholarship on contextual justice in healthcare [21], wherein justice perceptions are informed by professional norms and situational realities rather than abstract notions of identical treatment. The finding has important theoretical implications: organizational justice frameworks must accommodate not merely whether distributions are identical but whether they are appropriately responsive to legitimate contextual variation.

Notably, the operationalization of equity differs from Western conceptualizations in important ways. While participants recognized needs-based, meritocratic, and seniority-based frameworks as individually coherent, they experienced distress not from the choice of one framework but from inconsistent application. This suggests that in Saudi healthcare contexts, the most critical fairness dimension may be consistency and explicit framework articulation rather than which specific framework is chosen. When managers clearly communicate which principle guides allocation (“Today I’m prioritizing patient safety, so complex patients go to most experienced nurses”) and apply it consistently, nurses experience greater fairness than when managers apply principles inconsistently or opaquely.

### 4.3. Justice-Burnout Pathways: Multi-Level Analysis

The pathway from perceived allocation injustice to burnout articulated by participants reflects mechanisms well established in occupational health psychology. Perceived inequity generates emotional stress (anger, resentment, betrayal) that, when sustained, produces emotional exhaustion. Unacknowledged effort compounds this exhaustion by eroding meaning and motivation, leading progressively to depersonalization and reduced professional accomplishment. Several participants explicitly described this trajectory, with their narrative accounts mapping onto the dimensions of the Maslach Burnout Inventory developed through decades of occupational health research [20].

Participants linked allocation injustice not only to personal burnout but to collective team dynamics and broader organizational outcomes. This systemic perspective suggests that allocation inequity functions as a stressor at multiple levels: individual (compromising nurse well-being and morale), interpersonal (damaging team cohesion and trust, generating resentment between favored and disfavored nurses), and organizational (impairing care quality and patient safety). This multilevel perspective deepens understanding of how organizational practices reverberate through healthcare systems to affect patient outcomes and confirms that allocation equity is a quality and safety issue, not merely a human resources concern, with direct implications for institutional performance.

### 4.4. Procedural Justice, Psychological Safety, and Voice Behavior

The emphasis participants placed on transparent, consistent, and respectful allocation processes, as well as their reported willingness to accept less favorable individual outcomes if processes were just, reflects the well-documented “fair process effect” whereby procedural justice dampens negative reactions to unfavorable distributive outcomes [22,23]. This effect is well documented in the organizational literature; the present findings confirm its presence in Saudi nursing contexts and suggest it may be particularly pronounced in cultures that emphasize respect for authority and hierarchical relational structures.

Importantly, participants’ reluctance to voice concerns about allocation, which they attributed to fear of retaliation and organizational dismissiveness, reflects low psychological safety as theorized by Edmondson [24]. Psychological safety exists when team members believe they can speak up about problems, ask questions, and share concerns without fear of punishment or humiliation. The data indicate that most participants lacked this safety with respect to allocation concerns. This creates a vicious cycle: concerns go unvoiced, management remains unaware of allocation problems, existing inequities persist and intensify, and nurses’ resentment accumulates silently until it manifests as burnout and turnover. Breaking this cycle requires deliberate leadership action: soliciting input, receiving concerns without defensiveness, demonstrating responsiveness through visible investigation and change, and establishing zero-tolerance policies for retaliation against those who raise concerns.

The retaliation chill effect documented here carries particular significance in the Saudi healthcare context. When organizations penalize nurses who voice allocation concerns, they institutionalize silence and resignation, compounding pre-existing cultural norms around hierarchy and deference that may already inhibit direct communication with authority figures [11]. To counteract this dynamic, leaders must explicitly frame fairness questioning as a professional duty rather than an act of insubordination, thereby simultaneously dismantling structural and cultural barriers to voice behavior.

### 4.5. Contextual and Cultural Considerations: Beyond Western Frameworks

While the core justice dimensions (distributive, procedural, interactional) appear universally relevant, their operationalization and salience are culturally informed and may differ in important ways from Western healthcare contexts. Participants consistently framed perceived mistreatment in relational and dignitary terms, describing how dismissive managerial conduct generated a profound sense of disrespect that extended beyond the numerical allocation itself. This pattern is consonant with cultural scholarship documenting the centrality of respect and honor in interpersonal relations across many Middle Eastern contexts, concepts rendered in Arabic as ikhtiraam and sharaf, although participants in this study did not use these specific terms explicitly [25]. When managers treated nurses without respect or dismissed their input, the sense of injustice was particularly acute and extended beyond concerns about workload distribution to concerns about professional dignity and human worth.

This finding suggests that culturally responsive approaches to promoting allocation justice in Middle Eastern healthcare must attend not only to rational–technical dimensions (clear policies, objective criteria) but also to relational and symbolic dimensions that communicate respect and acknowledge nurses’ professional expertise and human dignity. The term “respect” in Middle Eastern contexts encompasses not merely professional courtesy but explicit acknowledgment of one’s place in a relational hierarchy and acknowledgment of one’s inherent worth.

Additionally, the implicit concern with wasta (relationship-based influence) emerged subtly throughout the data. While not explicitly named by most participants, concerns about favoritism and preference for personally connected colleagues reflect concern that allocation reflected informal networks and relationships rather than professional criteria. In contexts where wasta (relationship-based influence) is a normative social phenomenon across many domains [11], healthcare organizations must explicitly establish and enforce norms that prioritize merit-based and need-based allocation over relationship-based preferences. Achieving this requires not merely policy articulation but sustained cultural change in what is understood as appropriate organizational decision-making, supported by visible leadership accountability.

### 4.6. Nurses’ Future Perspectives: Evidence-Informed Recommendations

Theme 6 offered concrete, evidence-informed proposals for systemic reform that warrant explicit discussion. Participants’ recommendations for rotation systems, objective acuity-scoring tools, and technology-assisted allocation (Subthemes 6a and 6b) align with emerging evidence from healthcare systems research demonstrating that structured, transparent allocation tools reduce favoritism perceptions and improve nurse satisfaction [21]. The critical caveat offered by participants—that technology increases visibility but cannot substitute for organizational commitment (P10), which is consistent with implementation science literature on the limits of technical solutions in culturally embedded organizations. Subtheme 6c (policy and culture change) reinforced that written policies, leadership training, safe reporting channels, and demonstrated responsiveness are interdependent requirements of a coherent equity commitment, not interchangeable options. Future research should examine whether the specific rotation and acuity-scoring models proposed by participants are operationally feasible within Saudi nursing workforce structures and whether their adoption is associated with measurable improvements in perceived fairness, nurse retention, and patient safety outcomes.

### 4.7. Institutional Safeguards and Their Limitations

The finding that formal policies and grievance mechanisms were either absent, inconsistently implemented, or perceived as unsafe highlights a critical institutional gap. Organizations may assume that written policies and accessible grievance channels are sufficient to ensure fair allocation. The present findings indicate otherwise: formal structures function only when consistently implemented by leadership, when employees trust that using available channels will yield genuine resolution rather than retaliation, and when organizational culture actively supports fairness as a core operating value. This underscores that institutional change requires aligned action across multiple dimensions, including policy articulation, leadership training, cultural change, and accountability mechanisms, rather than merely the creation of formal structures.

Top-down policy reform is insufficient without corresponding shifts in leadership behavior and organizational culture. Leaders must visibly model commitment to fairness, demonstrate responsiveness to concerns raised by staff, and hold themselves and colleagues accountable for equitable allocation practices. Without this alignment, formal policies risk becoming procedural rituals that experienced nurses recognize as organizational performance rather than genuine commitment to fairness.

### 4.8. Implications for Practice and Leadership

Promoting work allocation justice requires coordinated, evidence-informed action across multiple organizational levels. First, organizations should develop and implement clear written allocation policies that specify criteria, decision-making authority, appeals processes, and monitoring mechanisms, with active nurse involvement in their design. Second, leaders require training in organizational justice principles, culturally responsive communication, and transparent decision-making; performance appraisal frameworks should incorporate fairness metrics. Multiple, psychologically safe reporting channels—formal, informal, and anonymous, must be established so that nurses can raise concerns without fear of reprisal. Fourth, technology-supported allocation tools should be introduced to enhance objectivity without replacing sound managerial judgment. Finally, organizations must cultivate a culture that explicitly values fairness, transparency, and accountability, as these conditions together reinforce staff trust, professional engagement, retention, and sustained patient care quality.

### 4.9. Contributions to Nursing Knowledge

This study contributes to a limited qualitative literature on organizational justice within Saudi and broader non-Western healthcare contexts by demonstrating how perceived allocation inequity affects nurse well-being, burnout trajectories, and patient care quality. It identifies actionable organizational practices: transparent written policies, explicit communication of allocation rationales, psychologically safe grievance channels, and consistent leadership accountability. These practices collectively support fair work allocation. The findings confirm that procedural and relational dimensions of justice are at least as consequential as distributional fairness, and that established organizational justice frameworks retain explanatory utility in this context while requiring culturally informed adaptation to account for the salience of respect and relational hierarchy in Saudi nursing practice.

### 4.10. Strengths and Limitations

Several methodological features strengthen confidence in the findings. Purposive sampling across three facility types (government, private, specialized) captured variation in governance models and practice contexts that a single-site study would have missed. Thematic saturation, reached at interview 13 and confirmed across interviews 14 and 15, supports analytic credibility by indicating that sufficient variation in perspectives was captured across the participating Saudi nursing contexts. Triangulation across data sources, analysts, and methods, combined with member checking in which 11 of 12 purposively selected participants confirmed thematic plausibility, strengthened analytic credibility and supported the consensus-building process central to reflexive thematic analysis. At the same time, important limitations should temper how findings are read. The sample is small (n = 17) and geographically confined to Riyadh, limiting analytic transferability to healthcare contexts with substantially different staffing models, cultural norms, or urban-rural compositions. The exclusion of nurse managers, though deliberate to focus on frontline experience, means that organizational rationales for observed allocation practices remain unexamined; a full picture of allocation justice requires perspectives from both sides. The cross-sectional design captures perceptions at a single point in time, precluding examination of how justice experiences evolve across a nursing career or shift in response to organizational policy changes. Finally, while back-translation procedures were employed, subtle semantic nuances in justice-related concepts (adl, haq, ihsan) may not fully survive translation from Arabic.

### 4.11. Recommendations

The most consistent finding across themes is that process quality matters as much as distributional outcome. Participants (e.g., P12) described tolerating unfavorable assignments when managers explained the rationale; they could not accept the same allocations made without justification. This points to a straightforward behavioral change: leaders should routinely communicate the criteria underlying assignment decisions, even briefly, rather than treating allocation as an administrative act that requires no explanation. Participants also described a pervasive retaliation chill that suppressed concern-raising (P5, P6, P13). Leaders should actively solicit feedback through formal channels that staff perceive as genuinely anonymous and safe and must be seen to act on what they receive. For healthcare organizations, the absence of written allocation policies was cited by most participants (P7, P14) as the primary driver of suspicion and perceived favoritism. Developing and communicating explicit criteria, whether needs-based, meritocratic, or seniority-weighted, matters less than applying whichever framework consistently and transparently. Technology-assisted allocation tools were welcomed by participants (P7, P10) as a mechanism for reducing subjectivity, with the important caveat that systems alone cannot change organizational culture (P10: “Technology just makes it visible”). Any technological implementation must be paired with sustained leadership accountability. For policymakers, the burnout pathways described by participants—from perceived injustice to exhaustion, meaning erosion, and turnover intention—correspond directly to national workforce retention challenges. National nursing workforce policy in Saudi Arabia should incorporate measurable allocation equity standards alongside existing staffing ratio requirements, and commission multiregional follow-up research to determine whether the patterns identified in Riyadh are consistent across the Kingdom.

## 5. Conclusions

Across the 17 Saudi nurses interviewed at three Riyadh-based facilities, work allocation justice was experienced across three intersecting dimensions: distributive fairness, procedural transparency, and interactional respect. Participants consistently associated perceived inequities (arising from staffing constraints, leadership variability, and the absence of written policies) with emotional exhaustion, reduced organizational commit.

## Figures and Tables

**Table 1:** Participant demographic and professional characteristics (*n* = 17).

Characteristic	Category	n (%)	Details
Age (years)	25–34	8 (47%)	Mean: 34.5 (SD 7.2)
	35–44	6 (35%)	Range: 25–49
	45–49	3 (18%)	
Gender	Female	14 (82%)	
	Male	3 (18%)	
Education Level	Diploma	9 (53%)	From accredited nursing programs
	Bachelor’s Degree	5 (29%)	BSc in Nursing
	Master’s Degree	3 (18%)	MSc or specialized preparation
Years of Nursing Experience	2–5 years	5 (29%)	Early career stage
	5–10 years	8 (47%)	Mid-career stage
	10–15+ years	4 (24%)	Senior career stage
Clinical Specialty	Intensive Care	5 (29%)	Critical care settings
	Medical–Surgical	4 (24%)	General/acute ward nursing
	Emergency	3 (18%)	Emergency department
	Pediatrics	2 (12%)	Children’s nursing
	Operating Theatre	3 (18%)	Surgical services
Facility Type	Government Hospital	7 (41%)	University-affiliated teaching hospital
	Private Hospital	6 (35%)	Teaching hospital, urban
	Specialized Center	4 (24%)	Medical center, specialized services
Shift Pattern	Day (06:00–14:00 or 06:00–15:00)	7 (41%)	
	Evening (14:00–22:00 or 15:00–23:00)	5 (29%)	
	Night (22:00–06:00 or 23:00–07:00)	4 (24%)	
	Rotating	1 (6%)	
Allocation Inequity Experience	Experienced inequities	15 (88%)	Personal direct experience
	No reported inequities	2 (12%)	Generally equitable units
Weekly Working Hours	40–44	12 (71%)	
	44–48	5 (29%)	Standard full-time arrangements
Saudi Nationality	Yes	17 (100%)	Inclusion criterion

**Table 2:** Major themes and subthemes emerging from the data analysis.

Theme	Subtheme	Description
Theme 1: Understanding Work Justice	Fairness Definitions	Nurses conceptualize fairness as contextual equity rather than identical equality; acknowledge need for differentiated allocation based on patient complexity, nurse capability, and circumstances
	Procedural Justice	Emphasis on transparent, consistent, rational decision-making processes; importance of managers explaining rationales; impact of process fairness on overall justice perceptions
	Justice Models	Participants articulate multiple frameworks (needs-based, meritocratic, seniority-based); tensions when inconsistently applied; variation in which framework is perceived as most just
Theme 2: Personal Experiences	Workload Disparities	Concrete experiences of unequal patient assignments; patterns of relationship-based, punitive, or specialty-based inequities; sustained resentment and demoralization
	Recognition Patterns	Inequitable distribution of feedback and acknowledgment; selective appreciation based on personal relationships; compounds allocation injustice perception
Theme 3: Influencing Factors	Staffing Levels & Constraints	Understaffing sometimes legitimately constrains equal distribution; however, chronic understaffing sometimes used to justify systemic inequities; importance of transparency about constraints
	Patient Acuity & Complexity	Legitimate variation in patient conditions should inform allocation; concerns about consistency and objectivity of acuity assessments
	Leadership Styles	Variation in managers’ approaches (soliciting input/explaining vs. unilateral/dismissive); impact on trust and justice perceptions despite objective allocation fairness
	Informal Peer-Based Balancing	Nurses develop informal agreements offsetting manager-imposed inequities; unit culture and peer relationships shape allocation experiences
Theme 4: Psychological & Professional Impacts	Job Satisfaction & Burnout	Perceived injustice linked to lower satisfaction and higher burnout symptoms; explicit articulation of burnout pathways (fatigue → meaning erosion → depersonalization)
	Organizational Commitment & Retention	Allocation inequity reduces organizational commitment and increases turnover intentions; loss of faith in institution
	Quality of Care	Perceived inequity compromises nurse attention, decision-making capacity, and patient safety; reduced cognitive resources under perceived injustice and overload
Theme 5: Institutional & Administrative Support	Allocation Policies	Absence of clear written policies; when policies exist, inconsistent implementation; lack of transparent criteria creates information void filled with suspicion
	Communication Channels	Limited opportunities to voice concerns; formal grievance mechanisms underutilized due to low confidence
	Grievance Mechanisms & Leadership Response	Concerns dismissed or resulting in retaliation; “retaliation chill” discouraging voice behavior; institutional failure to demonstrate responsiveness
Theme 6: Future Perspectives	Innovative Allocation Models	Rotation systems, objective acuity-based algorithms, technology-supported allocation; desire for systems constraining subjectivity
	Technology’s Role	Digital tools can increase transparency and reduce favoritism; however, requires organizational will and leadership commitment
	Policy & Culture Change	Need for explicit written policies, manager explanation of decisions, safe concern-raising channels, demonstrated responsiveness, organizational commitment to fairness

## Data Availability

Due to the sensitive nature of qualitative interview data and ethical constraints protecting participant confidentiality, original transcripts are not publicly available. Researchers may request anonymized data subsets (coding framework, thematic summaries with exemplar quotations, and selected thematic excerpts) by contacting the corresponding author.
